# Jenner Monument

**Published:** 1852-01

**Authors:** 


*Jenner Monument.*—We have received the Circular from the Committee in London, of which J. Connolly, M. D., is chairman. Subscriptions are now actively solicited throughout Great Britain and elsewhere, for a statue in bronze to be erected in London, as a *tribute from all nations*, to the memory of Dr. Jenner, for the discovery of Vaccination as a preventive of Small Pox, by which millions of human lives have been preserved from a loathsome death.

The subscription is limited to *one dollar*, so as to multiply the contributors.

Committees have been appointed for the U. S. as follows, viz:

John C. Warren, M. D., John Ware, M. D., James Jackson, M. D., *Boston;*
Geo. B. Wood, M. D., R. Dunglison, M. D., T. D. Mutter, M. D., *Philadelphia;*
Martyn Paine, M. D., Horace Green, M. D., Chas. A. Dee, M. D., *N. York.*

To either of these gentlemen, subscriptions of *one dollar* for each subscriber may be remitted, and it is proposed to solicit subscribers not only among the profession, but generally from all who appreciate the object, by agents appointed for the purpose, to be selected by these committees.

